# A Non-Coding Genomic Duplication at the *HMX1* Locus Is Associated with Crop Ears in Highland Cattle

**DOI:** 10.1371/journal.pone.0077841

**Published:** 2013-10-23

**Authors:** Caroline Tina Koch, Rémy Bruggmann, Jens Tetens, Cord Drögemüller

**Affiliations:** 1 Institute of Genetics, Vetsuisse Faculty, University of Bern, Bern, Switzerland; 2 Interfaculty Bioinformatics Unit, University of Bern, Bern, Switzerland; 3 Institute for Animal Breeding and Husbandry, Christian-Albrechts-University Kiel, Kiel, Germany; Innsbruck Medical University, Austria

## Abstract

Highland cattle with congenital crop ears have notches of variable size on the tips of both ears. In some cases, cartilage deformation can be seen and occasionally the external ears are shortened. We collected 40 cases and 80 controls across Switzerland. Pedigree data analysis confirmed a monogenic autosomal dominant mode of inheritance with variable expressivity. All affected animals could be traced back to a single common ancestor. A genome-wide association study was performed and the causative mutation was mapped to a 4 Mb interval on bovine chromosome 6. The H6 family homeobox 1 (*HMX1*) gene was selected as a positional and functional candidate gene. By whole genome re-sequencing of an affected Highland cattle, we detected 6 non-synonymous coding sequence variants and two variants in an ultra-conserved element at the *HMX1* locus with respect to the reference genome. Of these 8 variants, only a non-coding 76 bp genomic duplication (g.106720058_106720133dup) located in the conserved region was perfectly associated with crop ears. The identified copy number variation probably results in *HMX1* misregulation and possible gain-of-function. Our findings confirm the role of *HMX1* during the development of the external ear. As it is sometimes difficult to phenotypically diagnose Highland cattle with slight ear notches, genetic testing can now be used to improve selection against this undesired trait.

## Introduction

Isolated congenital malformations of the external ear are known in humans and different animal species [1]–[3]. In humans several responsible genes have been identified, most of them from the homeobox gene family [4].

Almost one hundred years ago, cattle showing crop ears were reported for the first time [5]. Cattle with crop or notched ears show variably nicked ears sometimes combined with deformed ear cartilage and shortened pinnae. Isolated external ear anomalies are known in Scottish breeds such as Ayrshire and Highland cattle, and in Irish Dexter cattle [5]–[9]. Possible correlations between milk production and crop ears in Ayrshire cattle have been excluded [7]. So far, little is known about the etiology and probable genetic causes of crop ears. Monogenic autosomal dominant inheritance with variable expressivity has been suggested by several authors [5]–[8]. Another probably dominantly inherited non-syndromic ear anomaly was reported in British Jersey cattle, in which the notch is located at the lower edge of the ear and looks like the artificially placed “underbite” mark by breeders [10]. Among goats, the Lamancha breed is easily recognizable by their very short ear pinnae. The missing or reduced external ears are a specific characteristic of this dairy breed [11]. Taken together, the underlying genetics of isolated ear anomalies in ruminants are still unknown. In dogs, a quantitative trait locus for floppy versus erect ears was recently mapped on canine chromosome 10 by genome-wide association [12].

In Switzerland we noticed the recurrent incidence of crop ears in Highland cattle. Local regulations ban sires from breeding, if one parent has notched ears. The aim of the study was to develop a genetic test for selection purposes. Cohorts of affected and normal Highland cattle were collected to map the responsible locus within the cattle genome and subsequently to detect the causative mutation.

## Results and Discussion

### Crop ears in Highland cattle

**Figure 1:**
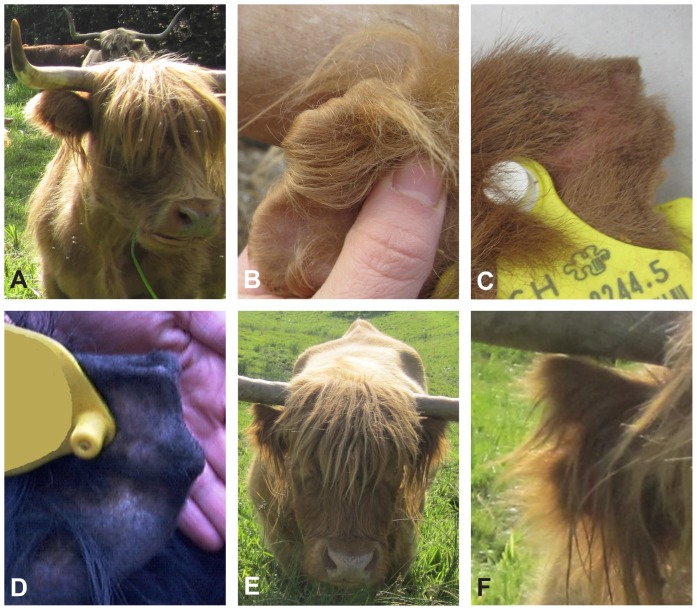
Crop ears in Highland cattle. (**A**) Normal ear, (**B,C,D**) mildly affected ears, note the slight notches (category 1), (**E**) severely affected cow; note the shortened ears and (**F**) the deep notches (category 2).

Crop ears in Highland cattle generally affect both ears more or less symmetrically, with a nick on the tip of the ear (Figure 1). Crop ears are already present at birth and occur both in male and female cattle. We classified the crop ear phenotype into 2 general categories of varying severity: in category 1 the notches are mild to moderate and the ear cartilage appears normal or is only mildly deformed (Figure 1B,C,D). Category 2 is defined by clearly shortened ears, large notches and prominent (and slightly caudally twisted) upper edges of the ear cartilage above the notches (Figure 1E,F). However, the phenotypic severity of crop ears varies greatly within both categories. A previous study in Highland cattle reported the presence of very short ears, almost rudimentary, and graded these into a separate third class [8]. Based on our observations we can neither confirm older reports that notched ears show more hair than normal ears, nor the presence of wart-like cartilage formations in crop ears [6], [8]. The Swiss Highland Cattle Society registers animals with visibly notched ears during their linear type evaluation [13], [14]. We collected blood or hair samples from 40 Highland cattle with notched ears and 80 with normal ears. Of these 40 cases, 39 belonged to the first and only one to the second category, respectively. None of the affected cattle showed extremely rudimentary ears. One cow (No. 26) had very large notches of 4 cm depth and the ears were markedly shortened (Figure 1E,F). We did not test the auditory capacity of the animals due to practical reasons. However, the farmers did not report any obvious hearing problems. This observation agrees with a previous report of crop ears in Highland cattle, in which no hearing abnormalities were noted [8].

### A dominantly inherited malformation

The available pedigree records of all affected cattle were analyzed for possible co-ancestry [14]–[16]. The earliest common ancestor is a sire born in 1943 and occurs about 8 to 12 generations ago (Figure S1). The pedigree can be explained by a dominant mode of inheritance, which is in accordance with previous studies [6]–[8]. Remarkably, multiple inbreeding loops are displayed indicating relatively close relationships between individuals within the Swiss Highland cattle population. This population is mainly derived from imported individual cattle and some intensively used Scottish sires during the last three decades. Interestingly, a common ancestor of affected animals in a previous report of crop ears in Highland cattle could be traced back to the same possible founder sire mentioned above [8].

Unfortunately, in our study the phenotype of the parents' ears could not be evaluated because some animals had already been slaughtered. Moreover, the farmers usually paid no attention to the ears or their statements were not reliable. Previous studies reported that the mating of two normal cattle might lead to offspring with crop ears [6], [8]. This was explained by possible incomplete penetrance and/or variable expressivity. Apart from the farmers' possible inattention, another explanation would be that the parents' ears were falsely judged to be normal, whereas in reality their notches were very small and therefore difficult to detect, even with palpation. Finally, since beef cattle usually reproduce via natural service, a possibly higher number of unclear paternities might also be an explanation.

### Mapping to a region on BTA 6 containing *HMX1* as candidate gene

**Figure 2:**
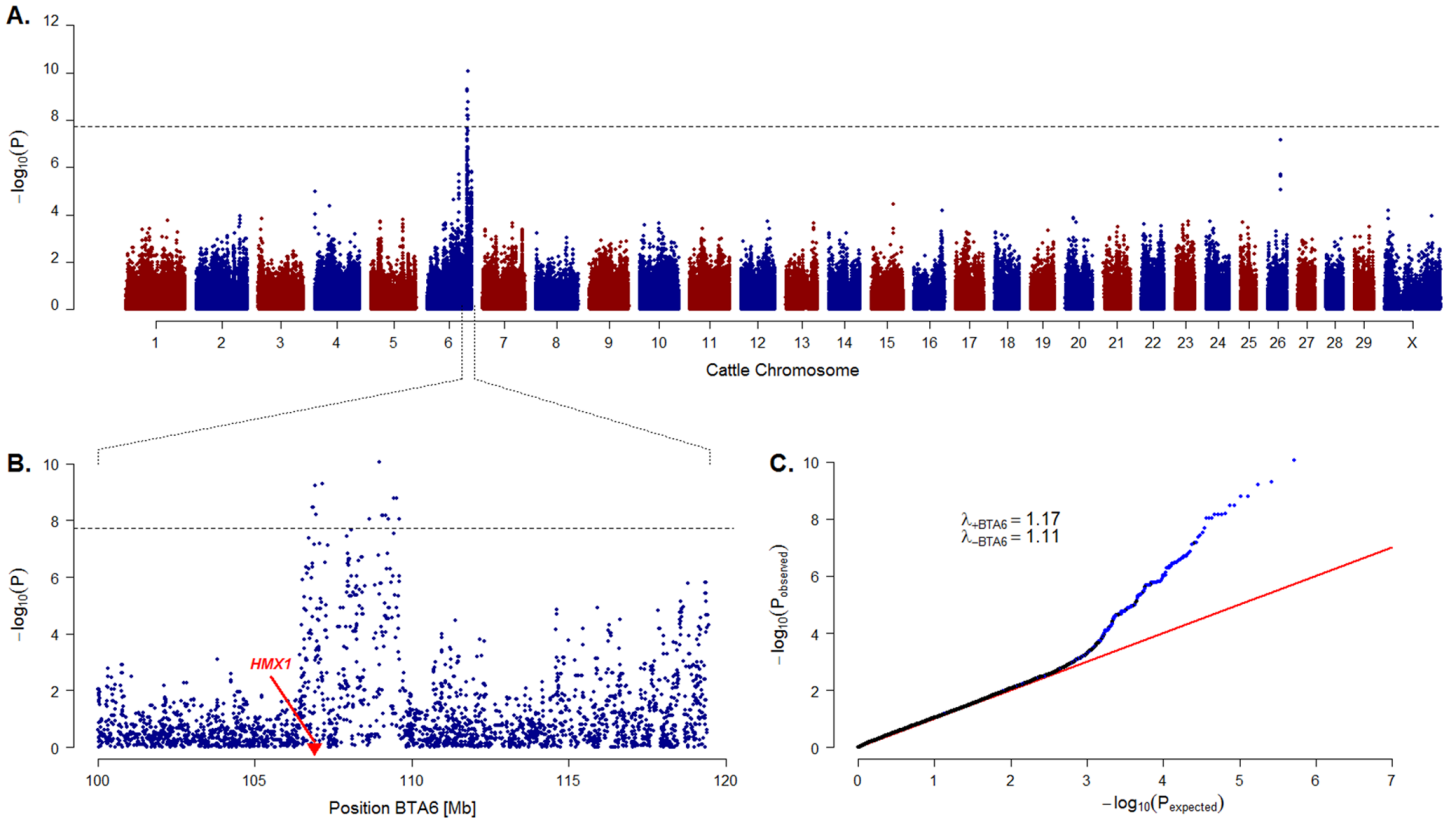
Results of the genome-wide association study. (**A**) Manhattan-plot showing the negative decadic logarithms of the raw P values obtained from the genotypic association test (χ^2^ test with 2 degrees of freedom). The dashed line indicates the Bonferroni-adjusted threshold for genome-wide significance (0.01 divided by 519,828 markers). (**B**) Detailed view of the significantly associated region of BTA 6. The position of the *HMX1* gene is highlighted in red. (**C**) QQ-plot reflecting the strong association signal on BTA6. The data points referring to SNPs located in the distal region of BTA6 shown in B are highlighted in blue. The genomic inflation factor λ is considerably smaller when excluding BTA 6 from the analysis (λ_-BTA6_) as compared to the genome-wide analysis including the chromosome (λ_+BTA6_).

A cohort of 32 affected and 36 control cattle were genotyped using illumina's bovine HD BeadChip containing 777,962 SNP markers. We performed a genome-wide association study (GWAS) and detected highly significantly associated markers (raw P values <10^−11^) in a single contiguous genomic region on cattle chromosome (BTA) 6 from 106 to 110 Mb (Figure 2A–B). The quantile-quantile-plot (Q-Q, Figure 2C) indicated an excess of small P values demonstrating the presence population stratification as to be expected regarding the structure of the analyzed cohort. However, this effect is largely explained by the strong association signal on BTA 6 as depicted in Figure 2C. The residual lambda value when excluding BTA 6 from the analysis is still comparatively high, which is probably due to the small sample size.

Within the associated 4 Mb region on BTA 6 a total of 64 genes are annotated and H6 family homeobox 1 (*HMX1*) was identified as striking functional candidate gene. In humans and mice, recessively inherited coding *HMX1* mutations lead to loss-of-function of this homeodomain containing transcription factor [17], [18]. A 26 bp deletion in the first exon of the *HMX1* gene is responsible for the human oculo-auricular syndrome, which is characterized by a particular cleft ear lobule in addition to eye malformations [17]. In mice, two independent *HMX1* mutations cause ventrally shifted ears with enlarged pinnae [18]. One mutation in the so-called *dumbo* mouse is a nonsense mutation affecting the first exon (*HMX1* ^dmbo^) and another mutation in the *misplaced ears* mouse is an 8 bp deletion within the second exon (*HMX1* ^mpe^) [18]. A recent study in *dumbo* rat mutants with a similar ear phenotype detected a genomic deletion affecting an ultra-conserved regulatory enhancer DNA sequence element several kb downstream of *HMX1*, which is required for the correct *HMX1* expression in craniofacial mesenchyme during development [19]. Given that the known coding and regulatory mutations at the *Hmx1* locus in human, rat, and mouse are all recessive, this raises interesting questions about the mechanism and the exact function of the bovine regulatory region duplication.

### Association of a non-coding duplication downstream of *HMX1* with crop ears

**Figure 3:**
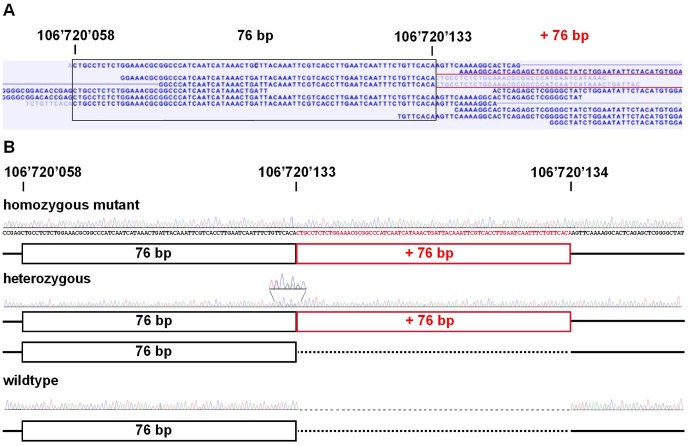
Mutation detection by whole genome re-sequencing. (**A**) Screenshot of the short read mapping against the reference sequence, note the red labeled reads indicating the genomic duplication. (**B**) Sanger sequencing confirmed the presence of the duplication; black box: 76 bp element in the reference sequence; red box: additional 76 bp duplication, note the enlarged region showing the heterozygous sequence using reverse PCR primer for sequencing.

**Figure 4:**
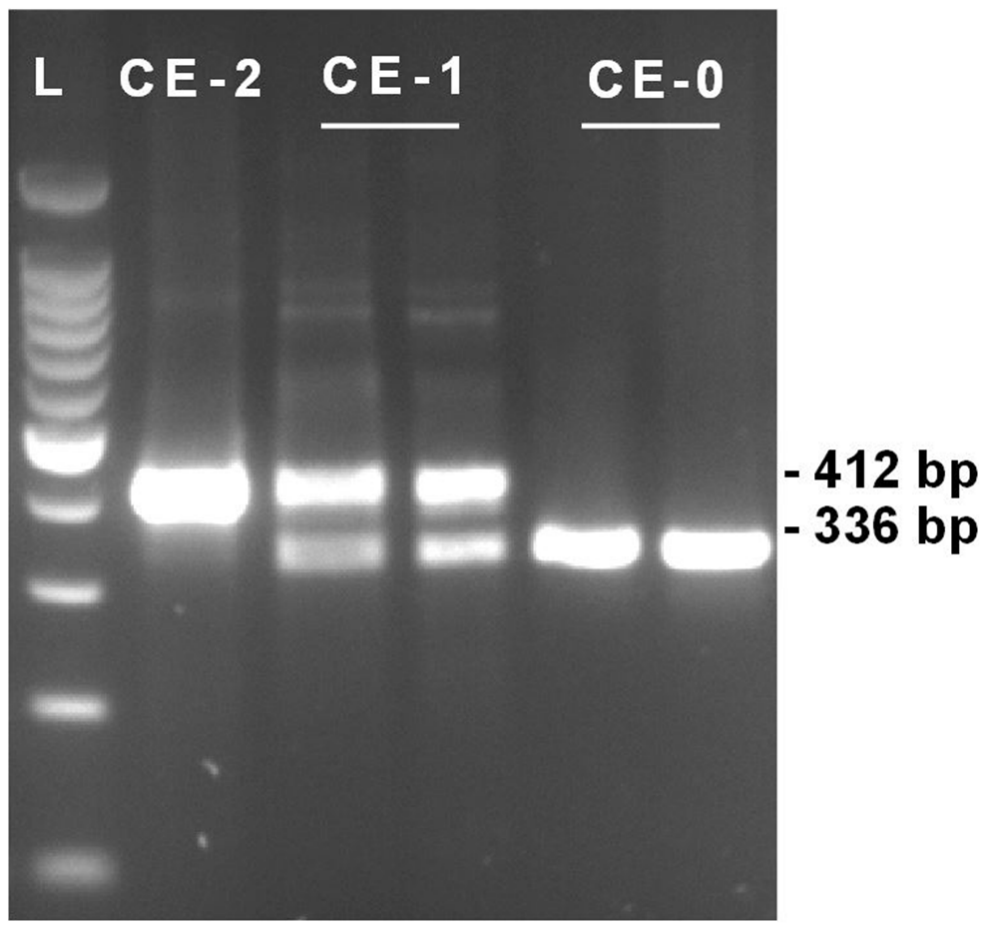
Agarose gel electrophoresis of diagnostic PCR for crop ear (CE) genotyping (L: Ladder 100 bp; CE-0: wild type control, CE-1: heterozygous affected, CE-2: homozygous mutant severely affected.

**Figure 5:**
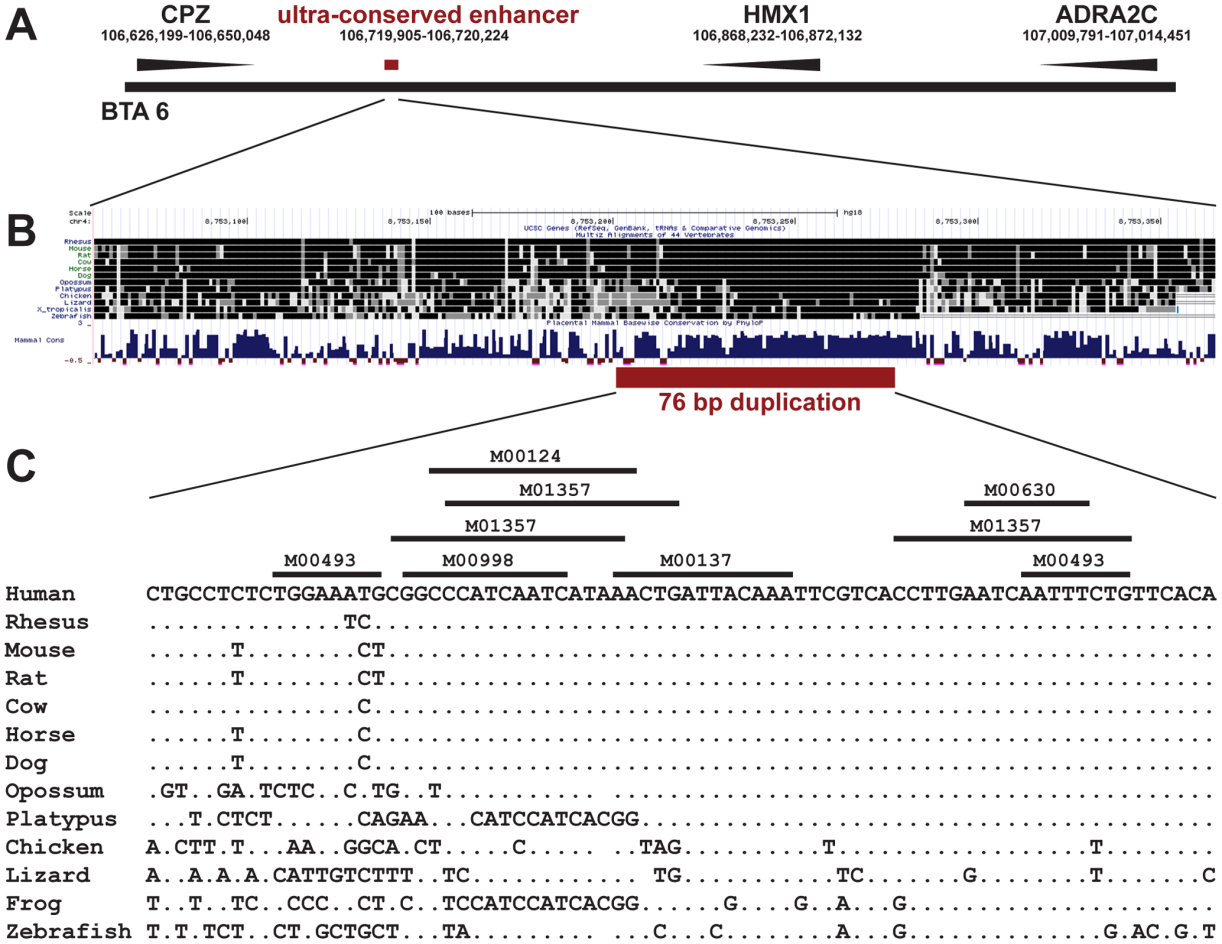
Comparative map of the HMX1 gene region on BTA 6. (**A**) Physical map position of HMX1 and flanking transcripts (UMD3 assembly). The ultra-conserved enhancer region is located 148 kb downstream of the coding region of HMX1. (**B**) Comparison and sequence conservation of the ultra-conserved enhancer region across different species (screenshot of the human UCSC genome browser [26]). (**C**) Multi-species genomic DNA sequence alignment showing the sequence conservation of the 76 bp duplication. The identifier of transcription factor matrices for potential transcription factor binding sites are shown above and were determined using Transcription factor Affinity Prediction (TRAP) software [27].

We selected a single severely affected cow (No. 26), which we assumed to be homozygous for the causative mutation based on the SNP chip data and performed whole genome re-sequencing using illumina's HiSeq2000 next generation sequencer. Analyzing the data from whole genome re-sequencing, we detected 6 non-synonymous DNA variants within the two coding exons of *HMX1* and two structural variants within the downstream highly conserved region by comparison to the bovine reference assembly (UMD3) (Table 1). A visual inspection of the paired-end read data also did not indicate any structural variation involving an annotated exon of flanking genes and the intergenic region up- and downstream of *HMX1*. By genotyping additional cattle with Sanger sequencing, all variants except one were shown to be not associated with the phenotype (Table 1). Furthermore, the 6 coding polymorphisms are obviously common variants as we identified an mRNA sequence entry containing the variant alleles (XM_003582354.1). Finally, only one of the two structural variants within the downstream conserved regulatory region, a 76 bp duplication (Figure 3), was shown to be perfectly associated with crop ears in Highland cattle (Table 1). For straightforward genotyping of this copy number variation (CNV) we designed a diagnostic PCR to verify the different genotypes by agarose gel electrophoresis (Figure 4, Table S1). As expected, the control animals showed a single band of 336 bp, the homozygous affected cow showed a single band of 412 bp and in heterozygous cattle both PCR products were detected. A total of 40 cases, and 80 controls from Highland cattle, and 144 animals of 23 different cattle breeds with unknown ear status were subsequently genotyped for this CNV (Table 1, Table S2). Only in Highland cattle showing crop ears at least a single copy of the duplication was present. Together with the reported findings in humans, mice and rats [17]–[19], we believe that the identified bovine *HMX1* associated CNV is very likely to be the causal mutation for crop ears. The affected ultra-conserved enhancer is located 148 kb apart of the coding region of *HMX1* (Figure 5A). The sequence of the 76 bp duplication is highly conserved (Figure 5B-C). *In silico* prediction revealed several transcription factor binding sites indicating the potential functional relevance of this region (Figure 5C). The CNV affects the similar conserved element as reported in *dumbo* rats [19]. Our findings support a functional role of this conserved DNA sequence element in regard to the expression of *HMX1* and they confirm the key role of *HMX1* during external ear development. Furthermore, this study provides an example of a non-coding but functionally important CNV in cattle [20]. A 6 kb genomic deletion, completely removing the highly conserved regulatory region downstream of the *HMX1* gene in rats, leads to the development of enlarged ear pinnae due to a loss-of-function of this enhancer [19]. Therefore, we speculate that the 76 bp duplication of crop ear affected Highland cattle may lead to a misregulation of *HMX1* expression and possible gain-of-function during embryonic development of the external ear.

**Table 1:** *HMX1* sequence variants.

HMX1	Variant on BTA 6 (UMD3 assembly)	cDNA	protein	genotype	Highland cattle cases controls		other breed controls
							
**highly**	g.106719992G>delG			G/G		78	88
**conserved**				G/delG	36	1	46
**region**				delG/delG	3		10
							
	**g.106720058_106720133dup**			**wt/wt**		**80**	**144**
				**wt/dup**	**39**		
				**dup/dup**	**1**		
							
**Exon 2**	g.106868441T>G	c.835A>C	p.M279L	T/T			
				T/G			
				G/G	2	4	
							
	g.106868626G>T	c.650C>A	p.P217Q	G/G			
				G/T			
				T/T	2	4	
							
	g.106868630T>A	c.646A>T	p.I216F	T/T			
				T/A			
				A/A	2	4	
							
**Exon 1**	g.106871834G>A	c.299C>T	p.P100L	G/G		2	32
				G/A	23		16
				A/A	5		1
							
	g.106871880G>C	c.253C>G	p.A85P	G/G			1
				G/C			15
				C/C	25	48	33
							
	g.106871897G>C	c.236C>G	p.G79A	G/G			7
				G/C		6	18
				C/C	25	42	24

Coding variants in the *HMX1* gene and structural non-coding variants in the *HMX1* associated highly conserved region. Note that the 76 bp duplication is perfectly associated with crop ears (bold face).

### Impact on selection against crop ears in Highland cattle

Even though crop ears are inherited dominantly, and may therefore be considered as easily eradicable from a breed, due to the long hairs and the extensive farming, smaller notches may not always be detected. This might explain the recurrent incidence of crop ears in the breed. Based on a small set of carefully phenotyped animals we could not confirm the presence of incomplete penetrance, as postulated by previous reports [5]–[8]. However, crop ears are variably expressed and extremely slight notches might have been overlooked in earlier studies. Crop ears may seem to be only blemishes, but mating two affected cattle can lead to calves with severe crop ears of category 2 (Figure 1E,F). It is conceivable that animals with such severely malformed external ears are not as able as others to locate noises due to their missing pinnae. In general, it is not advisable to mate two affected cattle. Severely affected animals with crop ears (CE) carrying two copies of the mutant allele, designated as CE-2, should be excluded from breeding. Furthermore, heterozygous animals carrying a single mutant allele (genotype CE-1) should, if at all, only be allowed to mate with cattle having the wildtype genotype, designated as CE-0. Our findings facilitate genetic testing and a controlled usage of possibly valuable Highland cattle with mild crop ears to minimize the loss of genetic diversity.

## Conclusions

We found a 76 bp genomic duplication downstream of the bovine *HMX1* gene associated with dominantly inherited crop ears in Highland cattle. We speculate that this segmental duplication within a highly conserved cis-regulatory element leads to a misregulation of *HMX1* expression and is probably causing this congenital malformation. The study supports the functional importance of *HMX1* expression during the embryonic development of the external ear. Our study clearly demonstrates the monogenic dominant inheritance of the crop ear mutation in Highland cattle with variable expressivity. This result enables a direct DNA-based selection against this undesired trait.

## Materials and Methods

### Ethics Statement

All animal experiments were performed according to local regulations. The cattle in this study were examined with the agreement of their owners. The study was approved by the “Cantonal Committee for Animal Experiments” (Canton of Bern; permits BE78/12).

### Animal Selection

We sampled 40 Highland cattle, which could be unambiguously phenotyped, based on having at least a mild notch in the ear cartilage. The 80 control Highland cattle were judged to have normal ears based on unremarkable physical examination. The complete cohort for this study consisted of 120 Highland cattle and 144 cattle of various other breeds with unknown ear status (Table S2). We collected EDTA blood or hair root samples from all Highland cattle.

### Genome-wide Association Study

Genomic DNA was extracted using QIAGEN DNeasy kit. We selected 32 cases and 36 controls for the GWAS. The animals were genotyped for the illumina BovineHD Bead array comprising a total of 777,962 SNP markers [21]. All samples have shown a sufficient call rate of ≥0.98. Markers were filtered for a maximum of 10% missing genotypes per locus and a minor allele frequency of ≥0.05 resulting in 19,376 and 238,169 SNPs to be omitted, respectively. Furthermore, markers with unknown genomic position or located on the Y-chromosome were excluded resulting in a final set of 519,828 SNPs. The GWAS was carried out using the R-package GenABEL [22] and applying a genotypic test for association (χ^2^ test with 2 degrees of freedom). The threshold of p≤0.01 for genome-wide significance was Bonferroni-adjusted to account for multiple testing (0.01/519,828 = 1.9×10^−8^).

### Whole Genome Re-Sequencing of an Affected Highland Cattle

We prepared a fragment library with 300 bp insert size and collected one lane of illumina HiSeq2000 paired-end reads (2×100 bp). We obtained a total of 237,905,693 paired-end reads or about 10× coverage. CLC Genomics Workbench version 6.0.2 (CLC Bio, Aarhus, Denmark) was used for read mapping against the cattle reference genome sequence (UMD3 assembly) [23].

### Genotyping

Primers for the amplification of the *HMX1* structural variants were designed with the software Primer3 [24] after masking repetitive sequences with RepeatMasker [25] (Table S1). We used Sanger sequencing to confirm the illumina sequencing results and to perform targeted genotyping for selected variants. For these experiments we amplified PCR products covering *HMX1* exon 1 and 2 and the ultra-conserved enhancer, respectively, using AmpliTaqGold360Mastermix (Life Technologies). PCR products were loaded on 2% agarose gels for visual inspection of band size. PCR products were directly sequenced on an ABI 3730 capillary sequencer (Life Technologies) after treatment with exonuclease I and shrimp alkaline phosphatase. We analyzed the sequence data with Sequencher 5.1 (GeneCodes).

## Supporting Information

Figure S1 Pedigree of the collected Highland cattle with crop ears. Note the multiple inbreeding loops and the earliest common ancestor appearing 8–12 generations ago. Only for the numbered animals the ear status was recorded. Affected animals are shown with black symbols, females are shown as circles and males as squares (+these animals were selected for SNP genotyping; *these animals appear twice).
(PDF)

Table S1 Primer sequences for the amplification of bovine HMX1 sequence variants. Positions refer to BTA 6 of the UMD 3 genome assembly.
(PDF)

Table S2 Number of genotyped animals in 23 different breeds.
(PDF)
