# Supplementary material for: A Non-Coding Genomic Duplication at the HMX1 Locus Is Associated with Crop Ears in Highland Cattle
Source: PLoS One. 2013 Oct 23;8(10):e77841. doi: 10.1371/journal.pone.0077841 (PMC3806818; doi:10.1371/journal.pone.0077841)
Supplement: Figure S1 — Pedigree of the collected Highland cattle with crop ears. Note the multiple inbreeding loops and the earliest common ancestor appearing 8–12 generations ago. Only for the numbered animals the ear status was recorded. Affected animals are shown with black symbols, females are shown as circles and males as squares (+these animals were selected for SNP genotyping; *these animals appear twice). (PDF) [file pone.0077841.s001.pdf]

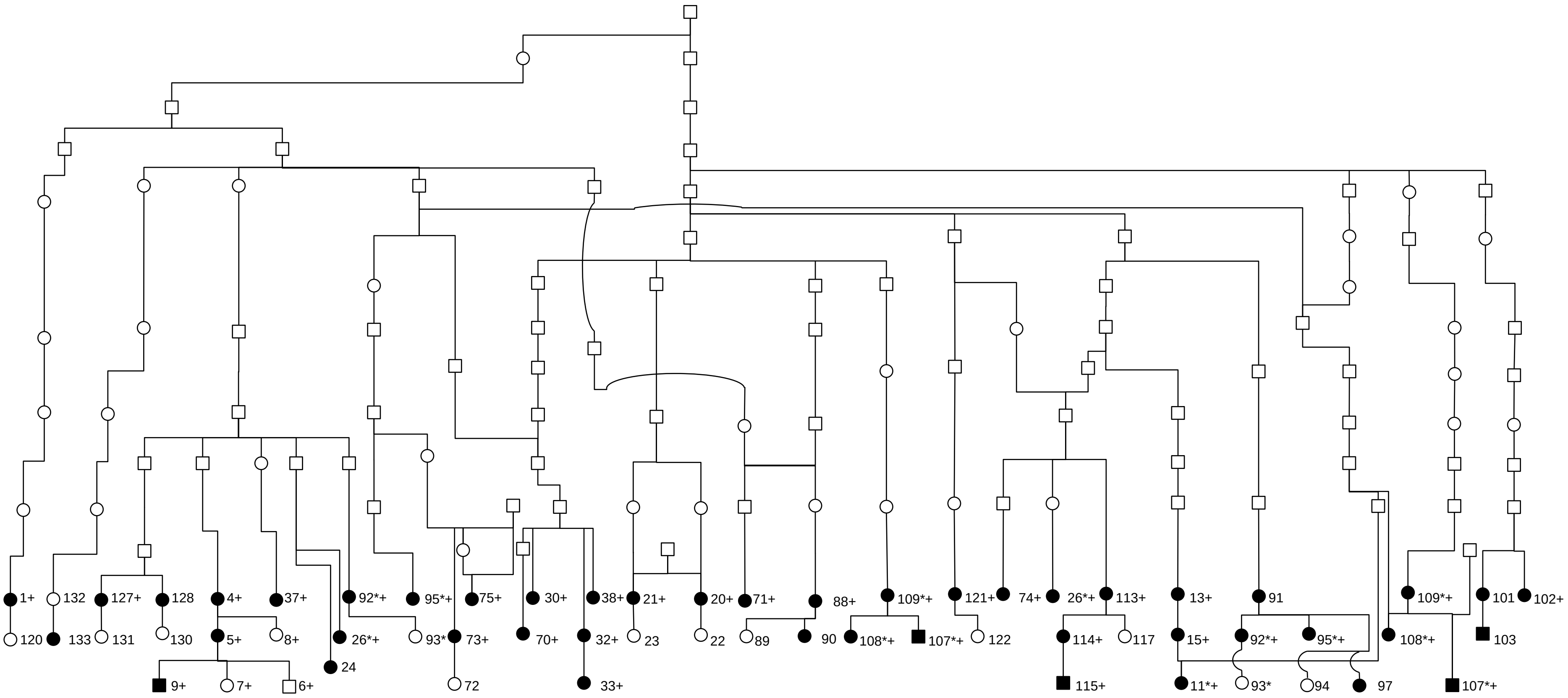

Supplementary Figure 1: Pedigree of the collected Highland cattle with crop ears. Note the multiple inbreeding loops and the earliest common ancestor appearing 8-12 generations ago. Only for the numbered animals the ear status was recorded. Affected animals are shown with black symbols, females are shown as circles and males as squares (**+**these animals were selected for SNP genotyping; *****these animals appear twice).
