# Supplementary material for: A Non-Coding Genomic Duplication at the HMX1 Locus Is Associated with Crop Ears in Highland Cattle
Source: PLoS One. 2013 Oct 23;8(10):e77841. doi: 10.1371/journal.pone.0077841 (PMC3806818; doi:10.1371/journal.pone.0077841)
Supplement: Table S1 — Primer sequences for the amplification of bovine HMX1 sequence variants. Positions refer to BTA 6 of the UMD 3 genome assembly. (PDF) [file pone.0077841.s002.pdf]

Table S1: Primer sequences for the amplification of bovine *HMX1* sequence variants. Positions refer to BTA 6 of the UMD 3 genome assembly.

	**Forward primer**		**Reverse primer**		**PCR product size (bp)**
HMX1	Start	Sequence 5'-3'	Start	Sequence 3'-5'	
Exon 1	106871605	CCATAGAGCCCACCACAGAC	106872770	TCCTTGCTGGACAGTGATTG	1166
Exon 2	106868022	TTACATTCTGGCCGATCCTC	106869009	GGGACTGTTTCCTTGCTGTG	988
Highly conserved region	106719573	TGCCCTGAGAACTTTCCATC	106720504	CAGCGACCCTCTCTCTCTTG	932/1008*
Primer for diagnostic PCR	106720053	CCGAGCTGCCTCTCTGGA	106720388	GAATCAGACAAGATCGCATCC	336/412*

*PCR product size in wild type animals/in mutants.
