# Supplementary material for: A Non-Coding Genomic Duplication at the HMX1 Locus Is Associated with Crop Ears in Highland Cattle
Source: PLoS One. 2013 Oct 23;8(10):e77841. doi: 10.1371/journal.pone.0077841 (PMC3806818; doi:10.1371/journal.pone.0077841)
Supplement: Table S2 — Number of genotyped animals in 23 different breeds. (PDF) [file pone.0077841.s003.pdf]

Table S2: Number of genotyped animals in 23 different breeds

**Breed**	**Number of animals**
Angus	10
Aubrac	1
Ayrshire	1
Belgian Blue	2
Belted Galloway	8
Braunvieh	11
Chianina	8
Dutch Belted	9
Eringer	17
Gelbvieh	1
Hereford	2
Holstein	24
Jersey	3
Limousin	1
Montbéliard	3
Nelore	2
Olore	2
Pinzgauer	8
Romagnola	2
Rotes Höhenvieh	13
Salers	1
Simmental	6
Tiroler Grauvieh	9
**Total**	**144**
